# Acceptability of screening for pregnancy intention in general practice: a population survey of people of reproductive age

**DOI:** 10.1186/s12875-020-01110-3

**Published:** 2020-02-20

**Authors:** Karin Hammarberg, Julie Hassard, Renee de Silva, Louise Johnson

**Affiliations:** 1 Victorian Assisted Reproductive Treatment Authority, Level 30, 570 Bourke Street, Melbourne, 3000 Australia; 2 School of Public Health and Preventive Medicine, Monash University, Level 4, 553 St Kilda Rd, Melbourne, 3004 Australia

**Keywords:** Preconception health, Preconception care, Health promotion, Pregnancy intention, General practice, Primary care

## Abstract

**Background:**

Optimal parental preconception health benefits reproductive outcomes. However, preconception health promotion is not routinely offered in primary health care settings to people of reproductive age. The aim was to gauge the planned preconception health behaviours and attitudes towards being asked about pregnancy intention by a general practitioner (GP) among people of reproductive age in Australia.

**Method:**

The research was conducted on a single wave of Australia’s first and only probability-based online panel, Life in Australia™. Members of the Life in Australia™ panel are Australian residents aged 18 years or over. All active members between the ages of 18 and 45 years were eligible to participate. Eligible panel members were invited to complete a survey about fertility and childbearing. Data were collected from 18 February to 4 March 2019.

**Results:**

In all 965 female and male members of Life in Australia™ aged between 18 and 45 years were invited to complete the survey. Of these, 716 (74.2%) agreed. Most respondents indicated that if they were planning to have a child they would try to optimise their preconception health by adopting a healthier diet (80%), seeing a GP for a health check-up (78%), reducing alcohol consumption (78% of those consuming alcohol), getting fitter (73%), and stopping smoking (70% of smokers). Three in four (74%) stated that they would not mind if their GP asked them about their pregnancy intentions.

**Conclusion:**

Findings suggests that routinely asking people of reproductive age about their pregnancy intentions and advising those who are planning pregnancy about what they can do to ensure optimal preconception health would be acceptable to most people and may improve reproductive outcomes.

## Background

There is now robust evidence about the importance of parental preconception health optimisation for the health of the pregnancy and the baby [1]. Parental exposure to potentially modifiable factors including poor nutrition, obesity, smoking, environmental toxins, and drugs and alcohol at the time of conception can adversely affect the health of the offspring at birth and increase their risk of non-communicable diseases including cardiovascular and metabolic conditions in later life [2].

General practitioners (GPs) and other primary health care professionals are ideally placed to opportunistically discuss pregnancy intention and promote pre- and inter-conception health optimisation with people of reproductive age. However, research shows that this is not routinely done and that health care providers’ perceived barriers for doing this include time constraints, lack of knowledge and resources, being unaware of preconception care guidelines, patients not appreciating being asked about preconception health, and competing preventive priorities [3–8].

The ‘One Key Question®’ concept developed in the United States recommends that primary health professionals routinely screen women of reproductive age for pregnancy intention by asking ‘Would you like to become pregnant in the next year?’ [9]. If the answer is no, this is an opportunity to mention the importance of reliable contraception to avoid unplanned pregnancy and refer the woman for contraceptive advice if she needs it. And, if the answer is yes or maybe, this opens the door for promoting the importance of preconception health optimisation and to suggest that women and their partner see their GP for a preconception health check before they start trying to conceive. In surveys of Maternal and Child Health Nurses and GPs most respondents indicated that they viewed the ‘One Key Question®’ concept as a useful tool for starting a conversation about preconception health [6, 7].

*Your Fertility* is an Australian government funded fertility and preconception health promotion program [10]. The aim of the program is to provide people of reproductive age with up-to-date, evidence-based, and accessible information about the factors that affect reproductive outcomes to allow them to make informed decisions about childbearing. The program also provides resources for health professionals to help them start conversations about reproductive health with their patients [11].

To inform the program, *Your Fertility* commissioned the *Social Research Centre* [12] to conduct a population-based survey gauging knowledge, attitudes and behaviours relating to fertility and preconception health among people of reproductive age in Australia. Here we report findings relating to planned preconception health behaviour and attitudes towards being asked about pregnancy intention.

## Method

### Study population

The study population were members of Life in Australia™, Australia’s first and only probability-based online panel [12]. The panel was established by the *Social Research Centre* in 2016 and is the most methodologically rigorous online panel in Australia. It exclusively uses random probability-based sampling methods and covers both online and offline population. Members of the panel are Australian residents aged 18 years or over who were randomly recruited via their landline or mobile telephone (rather than being self-selected volunteers) and consented to provide their contact details to take part in surveys on a regular basis [12]. Participants receive a AUD $10 to $15 reward for completing each monthly survey. Results from Life in Australia™ surveys are generalisable to the Australian population (see Appendix 1). All active members between the ages of 18 and 45 years were eligible to participate.

### Procedure

All eligible panel members were made aware of the opportunity to complete a survey about fertility and childbearing through the *Social Research Centre*’s regular communication. The survey was administered by the *Social Research Centre.* The methodology employed was a mixed-mode approach, using primarily online surveys supplemented with telephone surveys to include both the online and offline populations. Data collection was conducted from 18 February to 4 March 2019.

### Materials

The study-specific questionnaire was developed by the authors of the paper who have extensive clinical and research experience in the fields of reproductive health (nursing and midwifery) and health promotion (public education program management). It included questions about socio-demographic circumstances (age, country of birth, residential postcode, relationship status, and parenthood status); current health behaviours (smoking status, alcohol consumption, recreational drug use, exercise regularity, health status of diet, and weight [underweight, normal weight, a bit overweight, quite overweight]); parenthood aspirations (‘Do you want (more) biological children in the future’ and ‘How many children would you like to have’); likelihood of changing health behaviour in preparation for pregnancy rated on a 4-point Likert scale ranging from ‘Very Likely’ to ‘Not at all likely’ (take a multi vitamin [females only], eat healthier, see a GP for a health check-up, reduce alcohol consumption [drinkers only], stop smoking [smokers only] and lose some weight [only people who rated themselves as a bit or quite overweight]). Attitudes towards being asked about pregnancy intention were gauged with the question *‘How would you feel if your GP asked you ‘Would you (or your partner) like to become pregnant in the next year?’* where the response alternatives were ‘I wouldn’t mind’, ‘I would feel that it was inappropriate’, ‘I would appreciate it’, and ‘I would feel some other way’. The full questionnaire is available in Additional file 1.

### Statistical analysis

To correct for differences between the study population and the general population of people of reproductive age in Australia, and ensure the sample most closely represents the relevant Australian adult population, results were weighted to population benchmarks (see Additional file 2).

For the purpose of comparing levels of socio-economic advantage and disadvantage, respondents were assigned to quintiles based upon their residential postcode using the Index of Relative Socio-economic Disadvantage (IRSD). The IRSD is one of four indexes of socio-economic status developed by the Australian Bureau of Statistics (ABS) using information from their five-yearly Census. It distributes the population into five even quintiles denoting varying levels of disadvantage. A lower score (Quintile 1) indicates relatively greater disadvantage in general (e.g. more likely to include low income households, people with no qualifications, in low skill occupations). A high score (Quintile 5) indicates a relative lack of disadvantage in general.

Data were analysed in SPSS V25 using descriptive statistics. Gender and age group comparisons were made using Chi-square statistics and *p*-values < 0.05 were considered significant.

## Results

All active panel members (*n* = 965) were invited to take part and 716 (74.2%) completed the survey. The profile of respondents relatively closely matched the population for gender and residential location. However, there was some underrepresentation of people aged 18 to 25 and an overrepresentation of those aged 35 to 45 in the sample.

Table 1 shows the weighted profiles of respondents on a range of characteristics. While most respondents were Australian born, close to one in three were born overseas. Approximately one quarter of survey respondents belonged to Quintile 5, representing the least disadvantaged members of the population. Over two-thirds were in a relationship, most of whom were in a long-term heterosexual relationship. Respondents aged 18 to 24 years were significantly less likely to be in a long-term relationship than those who were older.

**Table 1:** Characteristics of respondents (weighted)

	Of all *N* = 716	Gender		Age group		
		Male (45%)	Female (55%)	18–24 years (14%)	25–34 years (37%)	35–45 years (48%)
Born in Australia (%)	71	71	71	73	70	70
Relative socio-economic disadvantage						
Quintile 1 - Most disadvantaged	15	16	15	10	17	16
Quintile 2	15	11	19	11	18	15
Quintile 3	23	29^	18*	26	23	22
Quintile 4	19	18	20	20	15	22
Quintile 5 – Least disadvantaged	27	26	28	32	26	25
Relationship status (%)						
In a relationship (opposite sex partner)	64	61	68	39*	65*	77^
In a relationship (same sex partner)	6	4	7	7	7	4
Not in a relationship	30	34	25	54^	28*	19*
Parental status and preferences (%)						
Have one or more children	42	35*	49^	1*	30*	76^
Want (more) biological children in the future	56	57	56	78	71	29*
Health and health behaviours (%)						
Current smoker (daily and occasionally)	17	21^	13*	17	16	19
Drink some alcohol most days	60	68^	53*	58	55	66
Use recreational drugs weekly or monthly	8	14^	5*	8	9	10
Do moderate exercise only monthly or less frequently	30	28	34	24	29	37
A bit or quite overweight	47	47	47	39	40	58
Unhealthy or very unhealthy diet	12	13	10	15	15	8

^ denotes significantly higher proportion and * significantly lower proportion. Where one proportion is higher or lower, it is significantly different to its one or two counterparts within the same subgroup

Around two in five respondents already had one or more children and more than half wanted a child or more children in the future. Respondents in the youngest group were less likely to have children and respondents in the oldest group were less likely to want children in the future than the two other groups. When asked how many children they would ideally like to have, including any children they already had, most (78%) wanted two or more children. Only 6% stated that they did not want children and 9% were unsure about how many children they wanted.

Self-reported health and health behaviours revealed that about one in six respondents were smokers, more than half consumed alcohol most days, one in 12 used recreational drugs at least monthly, almost one third rarely exercised and almost half considered themselves to be overweight.

Respondents were asked how likely they were to change some health behaviours in preparation for pregnancy. The proportions who stated that they were very or quite likely to change a range of health behaviours are shown in Table 2. Most respondents indicated they would try to optimise their health in one or more ways if they were planning to have a child now or in the future. Overall, females were more likely than males to state that they would reduce alcohol consumption, eat healthier and see their GP for a health check-up if they were planning pregnancy.

**Table 2:** Likelihood of changing behaviour if planning to have a child (% ‘Very likely’ and ‘Quite likely’)

Behavioural change	Of all (N = 716)	Gender	
		Male (45%)	Female (55%)
Take a multivitamin (females only) %	84	N/A	84
Eat healthier %	80	72	88^
See a GP for a health check-up %	78	71	85^
Reduce alcohol consumption (drinkers only) %	78	67	91^
Get fitter %	73	72	74
Stop smoking (smokers only) %	70	56	92
Lose some weight %			
Of those who reported being ‘a bit overweight’	78	77	79
Of those who reported being ‘quite overweight’	84	73	91

^ denotes significantly higher proportion

Responses to the question about how respondents would feel about being asked about their pregnancy intention are presented in Table 3. While most stated that they would not mind or would appreciate being asked this question, about one in four felt it would be inappropriate. The proportion who would feel the question was inappropriate was higher amongst those aged 18 to 24 years (33%) compared to those aged 25 to 35 years (19%), and among people who considered themselves to be quite overweight (39%) or a little overweight (29%), compared to those who reported a normal bodyweight (20%).

**Table 3:** Attitudes towards being asked about pregnancy intention (N = 716)

Response	%^#^
I wouldn’t mind	60
I would appreciate it	14
I would feel it was inappropriate	26
I would feel some other way	5

^#^ % is > 100 because respondents could select multiple responses. Where they had more than one opinion, this was because they indicated they would not mind and would appreciate it

## Discussion

This population-based study made two significant findings that can inform GPs approach to opportunistically asking their patients who are in the reproductive age range about their pregnancy intentions and promoting preconception health optimisation. Firstly, most women and men indicated that they would make positive health behaviour changes in preparation for pregnancy. This suggests that they are aware of the importance of preconception health optimisation and are likely to appreciate being supported by their doctor in their efforts to improve their health in the context of pregnancy planning. Secondly, most participants, both women and men, appeared positive about being asked about their pregnancy intentions. This is encouraging because it shows that asking a non-judgemental screening question like the ‘One Key Question®’ would in most instances be well received and allow GPs to provide relevant advice depending on the answer. However, it is noteworthy that two groups were less likely than others to appreciate being asked about pregnancy intention; respondents aged less than 25 years and respondents who reported being a little or quite overweight. It may be that people in their early 20s do not perceive a question about childbearing to be relevant to them as they are not planning to have children until later in life. For people who are overweight the question may be perceived as intrusive and likely to lead to unwanted discussions about weight loss. Although they may not appreciate it, it is still important to ask young people and those who are overweight about their pregnancy intention to ensure that they have reliable contraception if they want to avoid pregnancy and can be encouraged and supported to optimise their health if they wish to have children.

The Royal Australian College of General Practitioners ‘Guidelines for preventive activities in general practice’ [13] state that ‘Every woman of reproductive age should be considered for preconception care’ and recommend that GPs assist their patients to ‘develop a reproductive life plan that includes whether they want to have children’. We suggest that this recommendation should be extended to also include men of reproductive age as their health and health behaviours also affect the health of offspring [14]. Although men report wanting to be involved in childbearing discussions, they feel that they do not have a voice on the topic because reproductive health is perceived to be women’s domain [15]. Including men in discussions about childbearing and encouraging them to be active participants in decision making and to optimise their health in preparation for parenthood would benefit them, their partner and future children.

One of the known barriers for providing preconception care for women is that they often do not consult their GP until after they have conceived [4]. And, in the case of men, they are less likely than women to seek primary health care [16] and rarely consult a GP in preparation for pregnancy [17]. This means that preconception health promotion needs to be offered opportunistically in any consultation with people of reproductive age. To this end the ‘One Key Question®’ concept appears to be an efficient and feasible way to establish pregnancy intention as it adds minimal time to a consultation. For those who indicate that they would like to conceive in the next 12 months, GPs can mention the importance of optimal parental preconception health and recommend that they and their partner make a separate appointment for a preconception health check. And, for those who do not wish to become pregnant, asking about pregnancy intention offers the opportunity to establish contraceptive needs to avoid unplanned pregnancy.

GPs also cite lack of resources as a barrier to promoting preconception health and indicate that the availability of preconception care checklists and patient brochures and handouts would help them do this more often [7, 8]. Informed by this evidence, the *Your Fertility* program has a range of resources relating to preconception health optimisation for women and men that GPs can direct their patients to. These are evidence-based, written in accessible language and easily found on the program’s website where they can be downloaded [10].

The strengths of this study include that the study population closely resembled the general population of people of reproductive age in Australia, the large sample, and the relatively high response rate. Furthermore, the study generated important evidence about men who have largely been neglected in reproductive health research. Study limitations are also acknowledged. While almost three quarters of the eligible population completed the survey, self-selection bias, where people interested in fertility and childbearing were more likely to participate than people who were not, cannot be ruled out. Also, as a brief survey, the findings do not provide in-depth understanding of respondents’ attitudes or planned behaviours. Lastly, in light of the well-established evidence about the intention-behaviour gap [18], we acknowledge that responses to questions about planned preconception health behaviour change may not translate to actual change.

## Conclusion

The findings of this study suggest that routinely asking people of reproductive age about their pregnancy intentions and advising those who are planning pregnancy about what they can do to ensure optimal preconception health would be acceptable to most people and this may improve reproductive outcomes.

## Supplementary information

**Additional file 1.** Life in Australia™ - Family and having children survey questions.

**Additional file 2.** Appendix 1 Weighting.

## Data Availability

The datasets used and/or analysed during the current study are available from the corresponding author on reasonable request.

## Abbreviations

ABS: Australian Bureau of Statistics
GP: General Practitioner
IRSD: Index of Relative Socio-economic Disadvantage

## References

[1] Stephenson J, Heslehurst N, Hall J, Schoenaker DAJM, Hutchinson J, Cade JE, Poston L, Barrett G, Crozier SR, Barker M (2018). Before the beginning: nutrition and lifestyle in the preconception period and its importance for future health. Lancet.

[2] Fleming TP, Watkins AJ, Velazquez MA, Mathers JC, Prentice AM, Stephenson J, Barker M, Saffery R, Yajnik CS, Eckert JJ (2018). Origins of lifetime health around the time of conception: causes and consequences. Lancet.

[3] Bateson D, Black K (2018). Pre-conception care: an important yet underutilised preventive care strategy. MJA.

[4] Goossens J, De Roose M, Van Hecke A, Goemaes R, Verhaeghe S, Beeckman D (2018). Barriers and facilitators to the provision of preconception care by healthcare providers: a systematic review. Int J Nurs Stud.

[5] Hammarberg K, Collison L, Johnson L, Nguyen H, Fisher J (2016). Knowledge, attitudes and practices relating to fertility among nurses working in primary health care. Aust J Adv Nurs.

[6] Hammarberg K, Taylor L (2019). Survey of maternal, child and family health nurses’ attitudes and practice relating to preconception health promotion. Australian Journal of Primary Health.

[7] Hogg K, Rizio T, Manocha R, McLachlan RI, Hammarberg K: Men’s preconception health care in Australian general practice: GPs’ knowledge, attitudes and behaviours. *Australian Journal of Primary Health* 2019, 10.1071/PY19069. 31554536

[8] Mazza D, Chapman A, Michie S (2013). Barriers to the implementation of preconception care guidelines as perceived by general practitioners: a qualitative study. BMC Health Serv Res.

[9] Stranger Hunter M (2017). Would you like to become pregnant in the next year? The one key question® initiative in the United States. IJBPE.

[10] Your Fertility: www.yourfertility.org.au

[11] Hammarberg K, Norman RJ, Robertson S, McLachlan R, Michelmore J, Johnson L (2017). Development of a health promotion programme to improve awareness of factors that affect fertility, and evaluation of its reach in the first 5 years. Reproductive Biomedicine & Society Online.

[12] Social Research Centre: www.srcentre.com.au

[13] RACGP: Guidelines for preventive activities in general practice, 9th edn. In*.* East Melbourne: Royal Australian College of General Practitioners; 2016.

[14] Shawe J, Patel D, Joy M, Howden B, Barrett G, Stephenson J: Preparation for fatherhood: A survey of men's preconception health knowledge and behaviour in England. PLoS One 2019, 10.1371/journal.pone.0213897. PMC6426231 30893380

[15] Grace B, Shawe J, Johnson S, Stephenson J. You did not turn up… I did not realise I was invited…: understanding male attitudes towards engagement in fertility and reproductive health discussions. Human Reproduction Open. 2019. 10.1093/hropen/hoz014. 10.1093/hropen/hoz014 PMC6573469 31218265

[16] Britt H, Miller G, Henderson J, Bayram C, Harrison C, Valenti L, Wong C, Gordon J, AJ P, Y P *et al*: general practice activity in Australia 2014–15. In*.* Sydney University Press, Sydney; 2015.

[17] O'Brien AP, Hurley J, Linsley P, McNeil KA, Fletcher R, Aitken JR (2018). Men's preconception health: a primary health-care viewpoint. Am J Mens Health.

[18] Davis R, Campbell R, Hildon Z, Hobbs L, Michie S (2015). Theories of behaviour and behaviour change across the social and behavioural sciences: a scoping review. Health Psychol Rev.

