# Supplementary material for: Acceptability of screening for pregnancy intention in general practice: a population survey of people of reproductive age
Source: BMC Fam Pract. 2020 Feb 20;21:40. doi: 10.1186/s12875-020-01110-3 (PMC7031940; doi:10.1186/s12875-020-01110-3)
Supplement: Supplementary file 1 — Additional file 1. Life in Australia™ - Family and having children survey questions. [file 12875_2020_1110_MOESM1_ESM.docx]

Life in Australia™ - Family and having children survey questions

Current relationship

*(ALL)

intro The following questions are asked on behalf of Monash University. They are interested in the opinions of Australians on family and having children.

To start with, we would like to ask some questions about you and your circumstances.

*(ALL)

SCREEN Firstly, how old are you today?

Age: <RANGE 18-97>

98. (Don’t know) / Not sure

99. (Refused) / Prefer not to say

*(cob_group<>1 AND SCREEN<46, AGED BETWEEN 18-45 AND NOT BORN IN AUS)

D1 If you were not born in Australia, for how many years have you lived in Australia?

Enter years: <RANGE 0-80>

97. Not applicable, I was born in Australia

98. (Don’t know) / Not sure

99. (Refused) / Prefer not to say

*(SCREEN<46, AGED BETWEEN 18-45)

Q1 Are you currently in a relationship?

(IF YES, PROBE TO CODE FRAME)

1. Yes, opposite sex partner

2. Yes, same sex partner

3. No

98. (Don’t know) / Not sure

99. (Refused) / Prefer not to say

*(Q1=1 OR 2, IN RELATIONSHIP)

Q1a Do you currently live with your partner (at least 50% of the time)?

1. Yes

2. No

98. (Don’t know) / Not sure

99. (Refused) / Prefer not to say

*(Q1=1 OR 2, IN RELATIONSHIP) [MULTI-RESPONSE]

Q2 How long have you been in a relationship with your partner?

Enter years: <RANGE 0-80>

Enter months: <RANGE 0-18>

98. (Don’t know) / Not sure ^

99. (Refused) / Prefer not to say ^

*(Q1=1 OR 2, IN RELATIONSHIP)

Q3 Which of the following best describes your relationship? Do you consider it to be…?

(READ OUT)

1. A long-term / permanent relationship

2. A short-term relationship that may develop into a long-term relationship

3. A short-term relationship with no plans for development

4. Other (please specify)

98. (Don’t know) / Not sure

99. (Refused) / Prefer not to say

*(Q1=1 OR 2, IN RELATIONSHIP)

Q4 What was your partner’s age at their last birthday?

*Please provide your best guess if you’re unsure of their exact age.*

Enter age: <RANGE 16-99>

98. (Don’t know) / Not sure

99. (Refused) / Prefer not to say

*(Q4=98 OR 99, UNSURE OF EXACT AGE)

Q4a Even if you can’t give the exact age, can you tell us which of the following age groups they are likely to fall into?

(READ OUT)

1. Less than 20 years

2. 20-29 years

3. 30-34 years

4. 35-40 years

5. Over 40 years

98. (Don’t know) / Not sure

99. (Refused) / Prefer not to say

Fertility

*(SCREEN<46, AGED BETWEEN 18-45)

FERINTRO The next section is about fertility. Fertility is the ability to conceive a baby. Healthy sperm and eggs are needed for a pregnancy to happen. We are interested in what you know about fertility.

*(SCREEN<46, AGED BETWEEN 18-45)

Q5 On a scale of 1 to 10 where 1 is not at all confident and 10 is the entirely confident, how confident are you about your understanding of…?

[RANDOMISE A-H AND RECORD IN VARIABLE]

a) The biology of reproduction (the process of conceiving a child)

b) Safe sex and prevention of sexually transmissible infections (STIs)

c) How to avoid unwanted pregnancy

d) How the menstrual cycle works

e) How to protect fertility

f) How weight affects fertility

g) How age affects fertility

h) How smoking affects fertility

1. Not at all confident

2.

3.

4.

5.

6.

7.

8.

9.

10. Entirely confident

98. (Don’t know) / Not sure

99. (Refused) / Prefer not to say

*(SCREEN<46, AGED BETWEEN 18-45)

Q6 How comfortable are you asking your health care provider about sexual and reproductive health matters?

(READ OUT)

[ROTATE 1-4 AND 4-1 ORDER AND RECORD IN VARIABLE]

1. Very comfortable

2. Comfortable

3. Uncomfortable

4. Very uncomfortable

98. (Don’t know) / Not sure

99. (Refused) / Prefer not to say

*(SCREEN<46, AGED BETWEEN 18-45)

Q7 At what age do you think fertility starts to decline for…?

[RANDOMISE A-B AND RECORD IN VARIABLE]

a) Women

b) Men

(READ OUT)

1. 20-24 years

2. 25-29 years

3. 30-34 years

4. 35-39 years

5. 40-44 years

6. 45-49 years

7. 50 years or more

8. Age doesn’t affect fertility

98. (Don’t know) / Not sure

99. (Refused) / Prefer not to say

*(SCREEN<46, AGED BETWEEN 18-45)

Q8 People who have fertility difficulties sometimes use assisted reproductive technology, like IVF, to conceive. If a woman has IVF treatment, what do you think is the approximate chance of having a baby after one attempt for women in these age groups?

a) 29 or younger

b) 30-34 years

c) 35-39 years

d) 40-44 years

*If you’re unsure, please provide your best guess.*

(READ OUT) (IF UNSURE, BEST GUESS IS FINE)

1. 50%

2. 35%

3. 30%

4. 25%

5. 20%

6. 15%

7. 10% or less

98. (Don’t know) / Not sure

99. (Refused) / Prefer not to say

Health and lifestyle

*(SCREEN<46, AGED BETWEEN 18-45)

HEALTHINTRO The next section is about your own health and lifestyle.

*(SCREEN<46, AGED BETWEEN 18-45)

Q9 How would you describe your weight?

(READ OUT)

1. Underweight

2. Normal

3. A bit overweight

4. Quite overweight

98. (Don’t know) / Not sure

99. (Refused) / Prefer not to say

*(SCREEN<46, AGED BETWEEN 18-45)

Q10 Which of the following best describes your smoking status? This includes cigarettes, cigars and pipes.

(READ OUT)

1. I smoke daily

2. I smoke occasionally (but not daily)

3. I don’t smoke now, but I used to

4. I’ve tried it a few times, but never smoked regularly

5. I’ve never smoked

98. (Don’t know) / Not sure

99. (Refused) / Prefer not to say

*(SCREEN<46, AGED BETWEEN 18-45)

Q11 Do you drink alcohol?

(IF YES, PROBE TO CODE FRAME)

1. Yes, 3 or more drinks on most days

2. Yes, less than 3 drinks most days

3. No

98. (Don’t know) / Not sure

99. (Refused) / Prefer not to say

*(SCREEN<46, AGED BETWEEN 18-45)

Q12 How often do you do moderate or vigorous physical activity or exercise? For example: tennis, jogging, cycling, swimming, etc.)

(READ OUT)

1. Daily

2. Less than daily

3. Weekly

4. Monthly

5. Less than monthly

6. Never

98. (Don’t know) / Not sure

99. (Refused) / Prefer not to say

*(SCREEN<46, AGED BETWEEN 18-45)

Q13 How often do you use recreational drugs (e.g. marijuana, cocaine)?

(READ OUT)

1. Weekly

2. Monthly

3. Less than monthly

4. Never

98. (Don’t know) / Not sure

99. (Refused) / Prefer not to say

*(SCREEN<46, AGED BETWEEN 18-45)

Q14 How would you rate your diet?

(READ OUT)

1. Very healthy

2. Healthy

3. Not healthy nor unhealthy

4. Unhealthy

5. Very unhealthy

98. (Don’t know) / Not sure

99. (Refused) / Prefer not to say

Fertility and reproduction plans

*(SCREEN<46, AGED BETWEEN 18-45)

PLANINTRO This section is about whether you have children or would like to have children in the future.

*(SCREEN<46, AGED BETWEEN 18-45)

Q15 Do you have any of the following children…?

(READ OUT ONE BY ONE)

*Please select all that apply.*

1. Biological children

2. Adopted children

3. Step-children

4. Foster children

98. (Don’t know) / Not sure

99. (Refused) / Prefer not to say

*(Q15=1-4, HAS CHILD(REN))

Q15a In total, how many of the following children do you have?

1. Biological children: <RANGE 1-20> [DISPLAY IF Q15=1]

2. Adopted children: <RANGE 1-20> [DISPLAY IF Q15=2]

3. Step-children: <RANGE 1-20> [DISPLAY IF Q15=3]

4. Foster children: <RANGE 1-20> [DISPLAY IF Q15=4]

98. (Don’t know) / Not sure

99. (Refused) / Prefer not to say

*(Q15a_1=1-20, HAVE AT LEAST ONE BIOLOGICAL CHILD)

Q16 How old were you when you had your child?

Enter age: <RANGE 15-70>

98. (Don’t know) / Not sure

99. (Refused) / Prefer not to say

*(Q15a_1=2-20, MULTIPLE BIOLOGICAL CHILDREN) [LOOP THROUGH THIS QUESTION FOR NUMBER OF CHILDREN SPECIFIED AT Q15a]

Q16a How old were you when you had your </ first / second / third / etc.> biological child?

Enter age: <RANGE 15-70>

98. (Don’t know) / Not sure

99. (Refused) / Prefer not to say

*(SCREEN<46, AGED BETWEEN 18-45)

Q17 Would you like to have [IF Q15=1 DISPLAY ‘another’, ELSE DISPLAY ‘a’] child in the future?

1. Yes

2. No

98. (Don’t know) / Not sure

99. (Refused) / Prefer not to say

*(SCREEN<46, AGED BETWEEN 18-45)

Q18 Ideally, how many children would you like to have (had) in total?

Enter number: <RANGE 1-20>

98. (Don’t know) / Not sure

99. (Refused) / Prefer not to say

*(Q1=1 OR 2, IN RELATIONSHIP)

Q18a How many children would your partner like to have (had) in total?

Enter number: <RANGE 1-20>

98. (Don’t know) / Not sure

99. (Refused) / Prefer not to say

*(SCREEN<46, AGED BETWEEN 18-45)

Q19 Realistically, how many children do you think you will have in total (including any children you already have)?

Enter number: <RANGE 0-20>

98. (Don’t know) / Not sure

99. (Refused) / Prefer not to say

*(SCREEN<46, AGED BETWEEN 18-45)

Q20 Are you or your partner currently pregnant?

1. Yes

2. No

98. (Don’t know) / Not sure

99. (Refused) / Prefer not to say

*(SCREEN<46, AGED BETWEEN 18-45)

Q21 If you plan to have a child or another child now or in the future, how likely would you be to do the following prepare for that?

[RANDOMISE A-G AND RECORD IN VARIABLE]

a) See a GP for a health check-up

b) Lose some weight

c) Stop smoking [DISPLAY IF Q10=1 OR 2]

d) Reduce alcohol consumption [DISPLAY IF Q11=1 OR 2]

e) Take a multivitamin [DISPLAY IF P_GENDER=FEMALES]

f) Eat healthier

g) Get fitter

(READ OUT)

1. Very likely

2. Quite likely

3. Not very likely

4. Not at all likely

98. (Don’t know) / Not sure

99. (Refused) / Prefer not to say

*(SCREEN<46, AGED BETWEEN 18-45)

Q22 How would you feel if your GP asked you ‘Would you [DISPLAY IF p_gender=MALES: ‘and your partner’] like to become pregnant in the next year?’ without you having brought up the subject?

(READ OUT ONE BY ONE)

*Please select all that apply.*

1. I would feel that it was inappropriate

2. I wouldn’t mind

3. I would appreciate it

4. I would feel some other way (please specify)

98. (Don’t know) / Not sure

99. (Refused) / Prefer not to say

*(p_gender=FEMALES AND SCREEN<46, WOMEN AGED BETWEEN 18-45)

Q23 How likely would you be to consider freezing your eggs to preserve your fertility and have children later in life?

1. Very likely

2. Quite likely

3. Not very likely

4. Not at all likely

5. I have already done this

98. (Don’t know) / Not sure

99. (Refused) / Prefer not to say

*(SCREEN<46, AGED BETWEEN 18-45)

INFERTINTRO Infertility, or difficulty achieving a pregnancy or carrying a pregnancy to term, affects about one in every six couples in Australia. Both males and females can have problems with fertility.

*(SCREEN<46, AGED BETWEEN 18-45)

Q24 Have you personally ever had any problems with fertility?

1. Yes

2. No

98. (Don’t know) / Not sure

99. (Refused) / Prefer not to say

*(SCREEN<46, AGED BETWEEN 18-45)

Q25 Have you and your (current or any previous) partner together been unable to get pregnant after 12 months or more of trying?

1. Yes

2. No

98. (Don’t know) / Not sure

99. (Refused) / Prefer not to say

*(Q25=1, INFERTILITY)

Q25a Did you (or your partner) seek any medical help or treatment?

(IF YES, PROBE TO CODE FRAME)

1. Yes, and were diagnosed with female and / or male infertility

2. Yes, but have not received a diagnosis (unexplained infertility)

3. No

98. (Don’t know) / Not sure

99. (Refused) / Prefer not to say

*(SCREEN<46, AGED BETWEEN 18-45)

CHECK If any of the previous questions made you upset or feel uncomfortable, we have some referral websites and numbers that may help:

- The Victorian Assisted Reproductive Treatment Authority (VARTA) have counsellors who are equipped to deal with any distress relating to fertility. You can visit their website: <https://www.varta.org.au/> or call them on 03 8601 5250.
- If you would like to talk to someone about how you have been feeling or have any concerns about your mental health, please visit: [www.lifeline.org.au](http://www.lifeline.org.au) or call Lifeline on 13 11 14.

1. Continue
