# Supplementary material for: Acceptability of screening for pregnancy intention in general practice: a population survey of people of reproductive age
Source: BMC Fam Pract. 2020 Feb 20;21:40. doi: 10.1186/s12875-020-01110-3 (PMC7031940; doi:10.1186/s12875-020-01110-3)
Supplement: Supplementary file 2 — Additional file 2. Appendix 1 Weighting. [file 12875_2020_1110_MOESM2_ESM.docx]

Appendix 1 Weighting

Sample surveys are a commonly used method for drawing inferences about a population based on responses from just a subset of it. To be able to draw such inferences, a probability sample is required – one in which each element of the population has a known, non-zero chance of selection. Since some units in the population may not have a chance of selection (for instance, persons without a telephone have no chance of selection for a telephone survey) and there may be different rates of response across unit characteristics, many sample surveys yield subsets that imperfectly cover their target populations despite the best possible sample design and data collection practices (Valliant *et al.,* 2013). In such situations, weighting can reduce the extent of any biases introduced through non-coverage.

For Life in Australia™, the approach for deriving weights generally consists of the following steps:

1. Compute a base weight for each respondent as the product of two weights:
   1. Their enrolment weight, accounting for the initial chances of selection and subsequent post-stratification to key demographic benchmarks
   2. Their response propensity weight, estimated from enrolment information available for both respondents and non-respondents to the present wave.
2. Calibrate the base weights so that they satisfy the latest population benchmarks for several demographic characteristics.

The first step is essential in providing the statistical framework necessary for making population inferences from a sample survey. The second step accounts for non-response bias and ensures that survey estimates are consistent with other sources. Each step will now be covered in turn.

#### Enrolment weights

At the time that Life in Australia™ was established, design weights for all panel members were calculated as the inverse of their probability of selection. This was derived using a single-frame approach (Best, 2010), where the probability of selection ($p$) was calculated as:

$$p=\frac{S_{LL}\times LL}{U_{LL}\times{AD}_{LL}}+\frac{S_{MP}\times MP}{U_{MP}}$$

where

- $S_{LL}$ is the number of survey respondents contacted by landline
- $U_{LL}$ is the population of the universe of landline numbers (based on the number of households and the estimated proportion of households with a landline)
- $LL$ indicates the presence of a landline in the respondent’s household (0 = no landline, 1 = landline present)
- ${AD}_{LL}$ is the number of in-scope adults in the respondent’s household
- $S_{MP}$ is the number of survey respondents contacted by mobile
- $U_{MP}$ is the population of the universe of mobile numbers (based on the number of adults and the average number of mobiles owned by each person)
- $MP$ is an indicator that the respondent has a mobile phone (0 = no mobile phone, 1 = mobile phone).

#### Adjustment for sample attrition

As is typical for a panel survey, not all members respond to all waves, some are retired from the panel and new members are recruited. To limit the impact of such events on the representativeness of estimates made from respondents, enrolment weights are adjusted through the use of propensity scores (Rosenbaum and Rubin, 1983). These were calculated through a logistic regression model predicting the likelihood of a panel member participating in the current wave, conditional on characteristics available for both respondents and non-respondents.

The model incorporated characteristics such as location (state / part of state), socio-economic indexes for areas, remoteness, age group, gender, citizenship status, speaking a language other than English, Aboriginal or Torres Strait Islander status, number of adults in the household, number of children in the household, household type, whether the respondent had worked in the last week, employment status, education, television viewing, digital affinity, caregiving, disability, volunteer status, phone status and sampling frame (landline or mobile).

To reduce the impact of very low or very high values, the predicted probabilities were collapsed into five classes (Cochran, 1968), with propensity scores assigned as the mean probability within each class. Base weights were then assigned as the product of the enrolment weight and the inverse of the propensity class score.

#### Calibrated weights

To ensure that estimates made from the dataset are representative of Australians aged 18 years to 45 years, the base weights were calibrated so that their distribution matched external benchmarks for the demographic parameters shown in the able below. The calibration method applied was generalised regression (GREG) weighting which uses non-linear optimisation to minimise the distance between the design and calibrated weights, subject to the weights meeting the benchmarks (Deville and Särndal, 1992).^[[1]](#footnote-1)^

Large differences in weights may lead to large variances in survey estimates, and so limiting these variations can improve the precision of estimates. The use of weighting constraints aims to reduce the variance at the same time as limiting increases in the bias. The method applied is incorporated directly in the calibration process. The impact of setting bounds on the weights is assessed by comparing the weighting efficiency (Kish, 1992) of calibrated weights for different constraints. Bounded weights are generally preferred when their efficiency is close to that of the unbounded weights.

Variables used for calibration, with population distributions and source

| Characteristic | Category | Population (%) | Source |
| --- | --- | --- | --- |
| **Age group / Highest level of education** | 18-24 Bachelor and above | 3.4 | ABS Table Builder Census 2016 and ABS Australian Demographic Statistics Dec 2017 |
|  | 18-24 Below Bachelor | 21.8 |  |
|  | 25-34 Bachelor and above | 15.3 |  |
|  | 25-34 Below Bachelor | 24.4 |  |
|  | 35-45 Bachelor and above | 12.7 |  |
|  | 35-45 Below Bachelor | 22.5 |  |
| **Location** | Greater Sydney | 22.5 | ABS Table Builder Census 2016 and ABS Australian Demographic Statistics Dec 2017 |
|  | Rest of NSW | 9.4 |  |
|  | Greater Melbourne | 21.7 |  |
|  | Rest of VIC | 5.2 |  |
|  | Greater Brisbane | 10.3 |  |
|  | Rest of QLD | 9.3 |  |
|  | Greater Perth | 8.5 |  |
|  | Rest of WA | 2.0 |  |
|  | Greater Adelaide | 5.2 |  |
|  | Rest of SA | 1.2 |  |
|  | ACT | 1.9 |  |
|  | Greater Hobart | 0.9 |  |
|  | Rest of TAS | 0.9 |  |
|  | NT | 1.2 |  |
| **Sex** | Female | 49.8 | ABS Table Builder Census 2016 and ABS Australian Demographic Statistics Dec 2017 |
|  | Male | 50.2 |  |
| **Telephone status** | Landline only / Dual user | 64.0 | ACMA Communications Report Dec 2016 |
|  | Mobile only | 36.0 |  |
| **Volunteer status** | Not volunteered | 80.5 | ABS Table Builder Census 2016 and ABS Australian Demographic Statistics Dec 2017 |
|  | Volunteered in last 12 months | 19.5 |  |

#### References

Best, J. (2010). First-stage weights for overlapping dual frame telephone surveys. Paper presented at the 65th annual conference of the American Association for Public Opinion Research, Chicago, IL, USA, 15 May.

Cochran, W.G. (1968). The effectiveness of adjustment by subclassification in removing bias in observational studies. *Biometrics*, 24, 295–313.

Deville, J.-C. & C.-E. Särndal. (1992). Calibration estimators in survey sampling. *Journal of the American Statistical Association*, 87(418), 376-382.

Kish, L. (1992). Weighting for unequal Pi. Journal of Official Statistics, 8(2), 183-200.

Lumley, T. (2004). Analysis of complex survey samples. *Journal of Statistical Software*, 9(1). <https://www.jstatsoft.org/article/view/v009i08> (accessed 26 September 2018).

Lumley, T. (2019) Survey: analysis of complex survey samples. R package version 3.35-1. <https://cran.r-project.org/package=survey>.

R Core Team (2019). R: A language and environment for statistical computing. R Foundation for Statistical Computing, Vienna, Austria. <https://www.R-project.org/>.

Rosenbaum, P. & Rubin, D. (1983). The central role of the propensity score in observational studies for causal effects. *Biometrika* 70(1), 41–55.

Valliant, R., Dever, J. A., & Kreuter, F. (2013). *Practical Tools for Designing and Weighting Survey Samples*. New York, NY, USA: Springer.

1. Refer to Lumley (2004, 2019) for more details on the implementation of GREG weighting in R (R Core, 2019) and to Valliant, Dever, and Kreuter (2013) for a more general treatment of design, weighting and estimation for sample surveys. [↑](#footnote-ref-1)
